# Supplementary material for: Effects of Elevated CO2 Concentration on Host Adaptability and Chlorantraniliprole Susceptibility in Spodoptera frugiperda
Source: Insects. 2022 Nov 7;13(11):1029. doi: 10.3390/insects13111029 (PMC9699368; doi:10.3390/insects13111029)
Supplement: Supplementary file 1 [file insects-13-01029-s001.zip › insects-1944405-supplementary.pdf]

Effects of Elevated CO_2 Concentration on Host Adaptability and Chlorantraniliprole Susceptibility in *Spodoptera frugiperda*

Supplement Table S1

Primers of detoxification enzyme genes used in the quantitative real-time PCR.

Identification	Primer Sequence (5′ → 3′)	
	Forward	Reverse
RPL10-insects	TGGGTAAGAAGAAGGCTACG	TGTTGATGCGGATGACAT
RPL13-JIA	GCCTTAACCCTGCTTTTGCTAG	GCTTCGCCCTTCAATACCTTC
CYP-19543	TGCCGCACCTACCACCAAA	TGCCCAAGTAAGAGCCAACG
CYP-17259	CTTACGCCTTTGTTCTCATCCG	CGTCAGGGCTCGCACTTCA
CYP-05919	ATGGCAAGTACGAATGGTGGA	CGGCTATCAGGCGAAAGAAA
CYP-22092	TGTTCGTGTTCTTCGCTGCTG	TCTCCTCCTGCGCCTTCCT
CYP-10853	GAAGTGTTCAGTGATCGTGGCG	AAACCGATTCCTCAAGGCTGT
CE-12593	AATGCCGAATCCTTGTCCG	TGATGATGGCTTCAGGGTGG

CE-20560	ACGACGAATGGCGGGAAGT	GGTGCCGTGGCGTAAGGTAT
CE-13095	TTCGTTACGGTCAACCACCC	GCTGCCTTCCACAACATCTAAAA
CE-21185	ACCTGTGCCTGAAGGGTCG	GTTGTTTGGGTGGTGGTGTTG
CE-12962-	AGATAGCATTCTGTTTGGACCCG	CCCAGCGATATGAGGCAGTTTT
ABC-01998	GCATCGCAGGTTAGGACAC	TTCACAGCAAACGGCACAG
ABC-02885	ACCTCATAGCACGATCACC	AAACGGCTTCCTTTACTGA
ABC-18240	AATGTCCACCCGTTTCTAC	TACTAACCATCGCACTGAC
ABC-22158	GAACGAGCATTCGACGACAAT	CGGCAAACCAGCTAACATCAG
UGT-18136	CCATTGCTCTACCCAGGGATT	TGGCACATCTGGTCCAAACA
UGT-17742	AAAAGGCAACCCTACCCAT	CTTAGCGGCCAGTTCACAA

Supplement Table S2

Toxicity effect of Chlorantraniliprole on 3rd larvae of *S.frugiperda* under elevated CO_2 and ambient CO_2.

Treatment	Concentration response regression equation	$\chi2$	*P*	LC_{50}(mg L^{-1}) 95% CI
F_AW_A	y=4.2826+0.7447x	51.9652	0.0001	9.19 (1.57 - 59.67)
F_EW_A	y=4.4479+0.7901x	39.6400	0.0001	5.00(0.32 - 9.55)

Note: 95% CI, 95% confident intervals; LC_{50}, the concentration of Chlorantraniliprole.

Supplement Table S3

Summary of the transcriptome sequencing data.

Sample	Total Clean Reads (M)	Clean Reads Q20 (%)	Clean Reads Q30 (%)	Clean Reads Ratio (%)	Total Mapping (%)
F_AW_A_1	41.99	97.1	92.59	95.83	69.25
F_AW_A_2	42.37	97.18	92.7	95.66	69.03
F_AW_A_3	42.47	97.13	92.56	96.92	67.51
F_EW_E_1	42.3	97.16	92.62	96.52	67.28
F_EW_E_2	42.18	97.29	92.95	96.25	68.49
F_EW_E_3	42.39	97.25	92.81	96.74	67.25

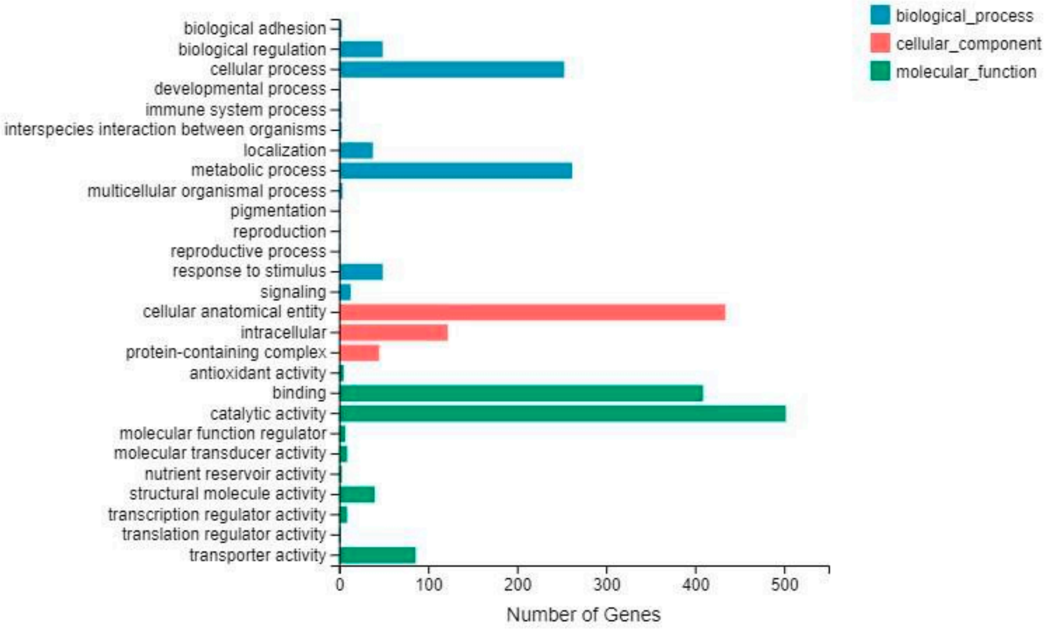

Supplement Figure S1. GO gene function classifications of DEGs in comparison groups. Gene function classifications of DEGs in F_AW_A versus F_EW_E. Note: The X-axis represents the number of genes annotated to GO entries and the Y-axis represents the GO functional classification.

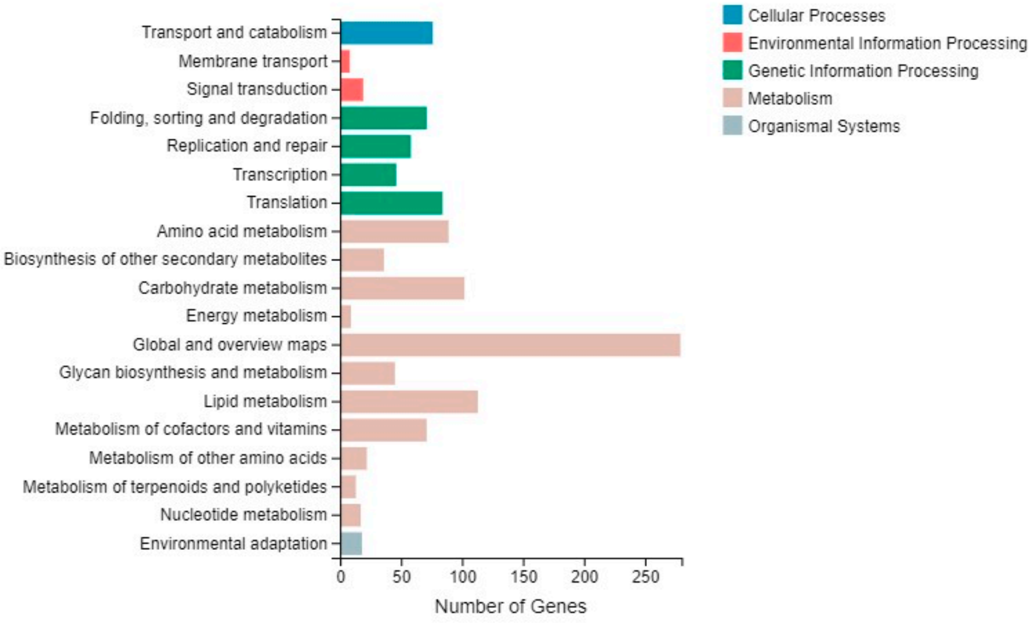

Supplement Figure S2. KEGG gene function classifications of DEGs in comparison groups. Gene function classifications of DEGs in F_AW_A versus F_EW_E. ***Note***: X-axis is the number of genes annotated to a particular KEGG Pathway category and Y-axis is the KEGG Pathway category.
